# *Helicobacter pylori-*Mediated Protection against Extra-Gastric Immune and Inflammatory Disorders: The Evidence and Controversies

**DOI:** 10.3390/diseases3020034

**Published:** 2015-03-27

**Authors:** Karen Robinson

**Affiliations:** Nottingham Digestive Diseases Biomedical Research Unit, School of Medicine, University of Nottingham, Nottingham NG7 2RD, UK; E-Mail: Karen.robinson@nottingham.ac.uk; Tel.: +44-115-823-1094; Fax: +44-115-846-8002

**Keywords:** *Helicobacter pylori*, extra-gastric manifestations, GORD, asthma, allergy, autoimmuity

## Abstract

A large number of studies link *H. pylori* infection with a reduced risk of developing extra-gastric conditions such as allergy, asthma, inflammatory bowel disease, coeliac disease and multiple sclerosis. The strength of the evidence for these protective associations is quite variable, and published studies often do not agree. This review article discusses some of the reasons for these discrepancies, and the difficulties faced when designing studies. Examples of some protective disease associations are described in detail, where the evidence is most abundant and thought to be more reliable. The most convincing of these are supported by published mechanistic data, for example with animal models, or incidence of disease exacerbation in humans following *H. pylori* eradication. Although controversial, this field is very important as the prevalence of *H. pylori* is decreasing throughout the world whilst many chronic diseases are becoming more common. These trends are likely to continue in the future, therefore it is important that we fully understand if and how *H. pylori* confers protection.

## 1. Introduction

*Helicobacter pylori* is a very common bacterial pathogen, which colonizes the mucosa of the human stomach. It is being intensively investigated as the main cause of peptic ulcer disease and gastric cancer, however these disease outcomes occur in a relatively small proportion of those infected (approximately 10%–20%) [1,2,3]. For the vast majority of people, there are no symptomatic indications that the bacteria are present in the stomach. This is despite gastric colonization generally persisting life-long from a very young age [4].

The prevalence of *H. pylori* is declining around the world, as fewer children are becoming infected [4,5]. The infection is virtually ubiquitous in developing countries (such as India and Mexico), but in many developed parts of the world (such as the USA, Scandinavia, Australia and the UK), the prevalence of the infection is now below 20%. As fewer than 5% of children in such regions are colonized, the decline will continue [2,3,6]. This is perceived as an unintended public health benefit arising from antibiotic usage in childhood since, as the prevalence of infection declines, cases of gastric and duodenal ulcers are also becoming less common [3]. Evidence is emerging, however, that adverse consequences may arise from a lack of exposure to *H. pylori.* This is hypothesised to be because humans have co-evolved with the bacterium over the last 60,000 years, and our physiology has developed taking account of its continual presence in the stomach [5,7]. When interactions with *H. pylori* are missing, this may lead to effects, such as defective development of the immune system, that increase the risk of developing diseases outside the stomach and duodenum. Many of these, such as asthma, inflammatory bowel disease, and autoimmune conditions, are becoming much more common as the prevalence of *H. pylori* has dropped [8,9]. They cause a great deal of pain and suffering, as well as becoming very substantial financial burdens on health care expenditure [10]. It is therefore extremely important to fully assess whether *H. pylori* infection plays a protective role, and determine how antibiotic eradication strategies should then ideally be managed in the light of this. Obviously it is necessary to eradicate the infection when patients have gastric symptoms, ulcers, or signs of pre-cancerous pathology. If *H. pylori* confers protection against other life-threatening diseases, however, it will be important to prevent exposure to this infection being completely lost from populations.

## 2. Weighing the Evidence for *H. pylori* Mediated Protection against Extra-Gastric Disorders

A huge number of published studies have now reported a correlation of *H. pylori* infection with lowered risk of developing extra-gastric diseases, and there is also some evidence for an influence on disease severity. These include gastro-oesophageal reflux disease and oesophagitis [5,11,12], asthma and allergy [13,14], several autoimmune disorders (including coeliac disease, systemic lupus erythematosus, rheumatoid arthritis and multiple sclerosis [15,16,17,18]), inflammatory bowel disease and irritable bowel syndrome [11,19]. It is intriguing to find so many reported protective associations against such widely varying conditions, and this raises questions about how these effects could possibly arise. It is suggested that the mechanisms might involve the reduction of gastric acid output [20]; *H. pylori-*mediated suppression of immune and inflammatory responses [14]; or modifications of the intestinal microbiota [5,21].

The idea that exposure to infectious organisms, particularly during childhood, is beneficial for health has been discussed for many years. Such exposure is thought to be important for the development of a healthy immune system, preventing the occurrence of conditions associated with an overactive immune response. The term “hygiene hypothesis” was first coined by David Strachan, in his paper showing that hay fever and eczema were less common in children from larger families. He proposed that the declining family size would result in a lack of protective unhygienic contact and cross-infection from other siblings [22]. This has recently been revised as the “Old Friends Hypothesis”, with further understanding that modernization has depleted access to many of the immunoregulatory stimuli that humans have co-evolved with [23]. Infections proposed to play a role in this include intestinal parasites, ectoparasites (such as ticks and lice), environmental bacteria, gut commensal organisms and also *H. pylori* [23,24,25,26,27,28]. Since exposure to some of these “Old Friends” and *H. pylori* is similarly associated with factors such as socioeconomic status, it is difficult to distinguish whether *H. pylori* itself could be a protective agent, or if it is merely a marker for some other as-yet unidentified factor.

Factors such as gender, age and social class influence *H. pylori* infection rates and gastro-duodenal disease risk [3]. These also affect the risk of developing many of the extra-gastric immune and inflammatory conditions listed above [29,30,31]. *H. pylori* is more common in those of older age, in males, and in populations of a lower socioeconomic status [3]. In contrast, many of the conditions that *H. pylori* is reportedly protective against are becoming more common in developed countries, especially those of higher socioeconomic status, and some are more common in females. Studies must therefore carefully control for such confounding influences and bias. Some associations are reported to be stronger with more virulent strains of *H. pylori*, for example those that possess the *cag* pathogenicity island (*cag*PAI) [12,32]. This provides some helpful clues about causal relationships because this *H. pylori-*specific virulence marker is not present in all strains. The best way to prove whether *H. pylori* is protective would perhaps be to administer an infection to volunteers and monitor them for systemic effects, e.g., by measuring inflammatory markers. It is only just becoming possible to deliberately infect humans with virulent *H. pylori* strains, and such studies have been restricted to healthy volunteers rather than those with underlying disease [33]. Many of the protective effects attributed to *H. pylori* could require infection from an early age, or over many years, and this makes such experimentation extremely difficult. Instead the literature to date are mostly based on epidemiological associations and, since *H. pylori* eradication therapy also affects other colonising bacteria, animal model studies currently provide the most reliable means to show cause and effect.

Unfortunately, in many cases the evidence for protective associations in humans is weak, contradictory and/or inconclusive. This tends to be because of constraints in study design. Most studies are cross-sectional, comparing the sero-prevalence of *H. pylori* in groups with and without disease. Wide variations in population size, and demographic differences between studies, mean that there are lots of conflicting reports. In general there is a real lack of longitudinal and mechanistic data. A systematic review published in 2014 by Smyk *et al.* [18] reported that 95 autoimmune conditions have been investigated as being influenced by *H. pylori.* Based on the strength of the evidence however, only four of these could be classified as having “probable” associations at best. All four were causative rather than protective associations.

Having discussed the barriers and difficulties in testing protective associations of *H. pylori* infection, the following sections will discuss the diseases for which the evidence is most abundant and more reliable.

## 3. Protection against Disorders of the Intestinal Tract

### 3.1. Gastro-Oesophageal Reflux Disease and Oesophagitis 

Gastro-oesophageal reflux disease (GORD) is a disorder arising from the retrograde flow of acidic stomach contents into the oesophagus. The development of gastro-oesophageal reflux is linked with several factors including obesity, gastric acid output, and consumption of fatty foods or drugs which may weaken the lower oesophageal sphincter [34,35]. Chronic mucosal damage from acid reflux can lead to a spectrum of symptoms, histopathological features and endoscopic findings, ranging from non-erosive to erosive oesophagitis, Barrett’s oesophagus, and oesophageal adenocarcinoma [35,36,37]. The incidence of GORD is increasing world-wide, in an inverse trend to the prevalence of *H. pylori* infection [38]. In Europe and the United States, where *H. pylori* is less common, GORD incidence is fairly stable but at the highest level (11%–23% and 17%–29% respectively). East Asia currently has a very low incidence of GORD (2%–9%), but this is increasing [34].

A relationship between *H. pylori* infection and GORD was first reported in 1998 [39], where the prevalence of *H. pylori* infection was significantly lower amongst patients with GORD. There were also negative associations with Barrett’s oesophagus and oesophageal adenocarcinoma. A number of subsequent studies supported the inverse relationship between *H. pylori* infection and GORD, showing that symptoms and endoscopic features developed or worsened after eradication of the infection [40,41]. They have also confirmed the protective associations with Barrett’s oesophagus and oesophageal adenocarcinoma [11,42]. It was proposed that a reduced acid output arising from *H. pylori-*induced corpus-predominant gastritis or pan-gastritis, plays a role in preventing damage to the oesophageal mucosa [43]. CagA positive strains, which cause more severe inflammation and are stronger acid suppressants, appeared have a greater protective effect [42]. In addition, eradication of *H. pylori* infection has been reported to result in increased production of ghrelin, a peptide hormone which stimulates appetite and promotes weight gain. Since obesity is a known risk factor for GORD and oesophageal cancer, suppression of ghrelin could be another mechanism by which *H. pylori* exerts an influence on the disease [35,44].

Although several studies provided evidence of *H. pylori* having an influence on GORD, others have refuted this and generated long-standing controversy within the field [45,46,47]. In particular, not all studies have shown an increased risk of GORD development in patients following *H. pylori* eradication therapy [41,48,49]. A number of issues could have contributed to the discrepant findings. Some studies, where all participants (both disease and control groups) were under clinical investigation for upper GI symptoms, may have unintentionally incorporated an element of selection bias. There has previously been a lack of agreement on the definition of GORD, and there are also differences in whether patients were assigned GORD status on the basis of symptoms and/or endoscopic findings. Another important source of discrepancy comes from failure to control for the pattern of *H. pylori-*mediated gastritis, or basing studies on populations where one gastritis pattern is more common [11,42,50]. In Western populations, infections are more commonly antral-predominant, which is associated with increased acid output [43,51]. In some Asian countries (including Japan), patients tend to have corpus-predominant gastritis and are consequently more likely to have a reduced gastric acid output [52]. When *H. pylori* is eradicated from these patients, gastric contents are likely to become more acidic and more damaging upon reflux into the oesophagus.

Awareness of these issues has been taken into account in a number of large-scale well-controlled multi-centre clinical studies and meta-analyses. These have confirmed that *H. pylori* eradication leads to increased risk of GORD, especially in Asian populations [50,53]. Others have successfully shown a negative association between the infection and Barrett’s oesophagus, as well as a stronger impact of CagA+ strains [11]. The controversies remain however, and in weighing the evidence, the recent Maastricht IV/Florence Consensus Report on the management of *H. pylori* acknowledges that GORD is less common amongst those who are infected, but concludes that eradication of *H. pylori* does not influence the severity of GORD [54].

### 3.2. Inflammatory Bowel Disease

Inflammatory bowel diseases (IBD), including ulcerative colitis (UC) and Crohn’s disease (CD), are chronic debilitating inflammatory disorders [55]. Potent immunosuppressant drugs are necessary to relieve the symptoms and control against severe recurrences, however these frequently fail to keep IBD in remission. CD can affect the entire length of the digestive tract (including the stomach), but is most commonly observed in the distal small bowel and the first section of the colon. In contrast, UC only affects the colon. Genetic susceptibility plays an important part in determining the risk of CD and UC development [56], however the prevalence of both is increasing and the reasons for this remain unclear. A large number of studies on IBD in adults and children have fairly consistently shown that *H. pylori* infection and its associated gastritis are less common in IBD cases (reviewed recently in [57]). A meta-analysis showed a significantly reduced risk of IBD when infected with *H. pylori* (relative risk 0.63) [58]. If *H. pylori* is really protective against IBD, then one might expect to find IBD development occurring after eradication of the infection. There are just two published reports on this, and both are concerned with CD. In 2001, Jovanovic *et al.* [59] reported that a patient given *H. pylori* eradication therapy due to dyspeptic symptoms developed Crohn’s disease three months later. Tursi [60] subsequently reported on two further patients who developed CD after receiving triple therapy for duodenal ulceration. There have been no further publications about this, and no reports at all on the development of UC following *H. pylori* eradication. Obviously it is possible that these patients might have developed CD regardless of any treatment for *H. pylori.* Several papers reporting the reduced incidence of *H. pylori* infection in IBD patients however, have warned of possible exacerbation following eradication therapy [61,62].

The possible mechanisms behind such associations are complex. There is published evidence for *H. pylori* being protective against IBD, as well as *H. pylori* colonization being inhibited by factors associated with the damaged mucosa and drug treatments for IBD [63,64]. It has been documented that amongst patients with CD-related gastritis, an unusually small proportion are *H. pylori-*positive [63]. Several mechanisms have been suggested for *H. pylori-*mediated protection against IBD. One possibility is that *H. pylori* infection of the gastric mucosa results in modification of the intestinal microflora, and immune responses to these organisms stimulate markedly less inflammation in the gut [65]. There is evidence from the Mongolian gerbil model that *H. pylori* infection causes alterations in the microbiota of the stomach and duodenum [66]. A long-term *H. pylori* colonization study in gerbils also showed that there was a change in the microbiota of the large intestine [21].

A few animal model studies have demonstrated that *H. pylori* exerts protective immunological effects against experimental colitis. In one study it was shown that oral doses of *H. pylori* DNA could substantially reduce the severity of dextran sulphate sodium induced colitis [67]. The protective mechanisms were proposed to be mediated via effects on dendritic cells, which were inhibited from producing proinflammatory cytokines after treatment with *H. pylori* DNA *in vitro*. In a second study from the same group [68], mice infected with *H. pylori* prior to induction of *Salmonella typhimurium* colitis, had markedly reduced levels of colonic inflammation compared to control animals that were not *Helicobacter-*infected. This was thought to be due to increased expression of interleukin-10 (IL-10), an anti-inflammatory and immunomodulatory cytokine, and reduced expression of proinflammatory IL-17, in the draining lymph nodes and mucosal tissues.

There is broad agreement that regulatory T cells (Tregs), a suppressive subtype of CD4^+^ T cells, play an important role both in IBD and in *H. pylori* infection. These cells can act in a bystander manner by secreting immunosuppressive cytokines such as IL-10 and transforming growth factor beta (TGFβ) to modulate inflammation, or they may act in an antigen-specific manner by a myriad of mechanisms (reviewed in [69]). *H. pylori* stimulates an enhanced Treg response, both in the gastric mucosa and peripheral blood. Tregs are thought to be important for maintaining persistent *H. pylori* colonization, via suppression of protective immunity, as well for limiting the severity of gastric inflammation so that disease outcomes do not usually result [70,71,72,73]. IBD patients (both CD and UC) tend to exhibit a marked deficiency in Tregs during relapses [74,75,76]. In a similar way to the suppressive effects on gastritis to prevent peptic ulceration, these cells are essential for controlling immune-mediated intestinal pathology [69]. IBD patients with the most severe disease, requiring surgical intervention, have the lowest numbers of Tregs in their peripheral blood [74]. Interventions to enhance the Treg response in CD patients have been tested in clinical trials, including the adoptive transfer of Treg cells [77,78], and administration of Treg-inducing infections (e.g., intestinal parasites [79]). It remains to be seen whether therapies can be developed and advanced, based on deliberate infection with *H. pylori*, or formulations of Treg-inducing *H. pylori* components.

## 4. Protection against Autoimmune Disorders

### 4.1. Coeliac Disease

Coeliac disease is an autoimmune condition that affects up to 1% of people in the developed world, but this is increasing [80,81,82]. Cross-reactive immune responses to a gluten protein found in wheat, result in inflammation and damage to the mucosa of the small intestine. This causes flattening of the villi and impaired absorption of nutrients, and is manifest in a variety of symptoms such as abdominal pain, mouth ulcers, anaemia, muscle cramps, joint pain and growth impairment. There is a very strong genetic component to disease risk [83,84]. Over the last few decades there have been a number of conflicting reports on associations with *H. pylori* infection. Some studies have shown that the prevalence of *H. pylori* is reduced in patients with coeliac disease [85,86,87,88], whereas others have found no differences [89,90,91] or even that it is increased [92]. These differing results could be explained by many of the same reasons stated in the previous sections: small sample sizes, determination of *H. pylori* status via different methodologies, and variable control for age, gender and socioeconomic status. This is important as coeliac disease is more common in females and in those of higher socioeconomic status, whereas *H. pylori* is more common in the opposite groupings [83,86]. A recent large-scale and well-controlled study compared the prevalence of *H. pylori* amongst 2,689 patients with coeliac disease and 127,619 patients with normal duodenal histology [17]. They found that the proportion infected amongst the coeliac disease patients was half that of the control group (OR 0.48; *p* < 0.0001).

The mechanisms by which *H. pylori* might protect against coeliac disease remain unknown and unexplored. It is possible that presence of the infection could affect the antigenicity of gliadin (via effects on gastric acid), or perhaps the Treg response induced by *H. pylori* modulates the autoimmune reaction. Animal models for coeliac disease do exist (reviewed in [93]), however these have not so far been used to investigate protective effects of *H. pylori.* There is no evidence for coeliac disease diagnosis arising directly after *H. pylori* eradication, and no published data show whether *H. pylori* infection status influences disease severity.

### 4.2. Multiple Sclerosis

Multiple sclerosis (MS) is an inflammatory demyelinating immune-mediated disorder which affects the central nervous system (CNS). Development of autoreactive T cell responses against CNS-derived antigens leads to an influx of Th1 and Th17 cells into the spinal cord and CNS [94,95]. These cells cause damage to the myelin sheath of neural axons, along with inflammation and degeneration of nerves [96]. Several epidemiological studies have reported a significantly lower prevalence of *H. pylori* infection amongst MS patients [97,98,99,100,101]. Additionally, two case control studies found that amongst MS patients, neurological disability was reduced in those with *H. pylori* [99,100]. In contrast, other studies have failed to find any association between *H. pylori* infection and MS [102], perhaps because of a positive association between *H. pylori* and a severe variant of MS, neuromyelitis optica (NMO) (reviewed in [18]).

To date, there is very little mechanistic evidence for a protective association between MS and *H. pylori.* There are no data concerning the impact of *H. pylori* eradication therapy on MS, and only one animal model study has been reported so far. Recently our group showed that prior *H. pylori* infection of mice inhibited the severity of experimental autoimmune encephalomyelitis (EAE), an animal model of MS [101], the most commonly used model for investigating human MS [103]. EAE was induced by immunization with the MOG_35-55_ myelin peptide, leading to an autoimmune response that mimics MS [103,104].

The hypothesis that *H. pylori* may be protective against MS and EAE is perhaps counterintuitive, since Th1 and Th17 cells, which play a role in disease pathogenesis [105], are also induced by the infection [106]. It has been shown that injection of mice with heat killed *H. pylori* bacteria and Freund’s incomplete adjuvant, however, is not sufficient to trigger EAE [107]. Our data showed that the numbers of CD4 cells in the spinal cords of infected animals was approximately half that of the uninfected controls. There was an extremely pronounced reduction in Th1 and Th17 cells, both in the spleen and in the spinal cord [101]. The balance between pro-inflammatory (Th1 and Th17) and anti-inflammatory (Treg) T cell subset responses is known to be important in the development and progression of MS [108,109]. Humans and animals infected with *H. pylori* have elevated IL-10-secreting Treg populations, therefore this suppressive T cell response may provide protective activity in a bystander fashion. In patients, *H. pylori* infection is associated with alterations in the profile of homing receptors expressed by peripheral blood T cells, directing their migration towards the inflamed gastric mucosa [70,110]. This includes an increase in the proportion of Tregs that express the chemokine receptor CCR6 [70]. CCR6 is a marker for Th17 cells and is important in modulating the balance between Treg and Th17 populations [111]. CCR6 plays an important role in EAE, since CCR6-deficient mice develop less severe disease [112] and are also less able to control EAE when it develops [113]. The infection may therefore alter the expression of chemokine receptors and integrins by T-effector or regulatory T cells, resulting in fewer T cells entering the CNS and inhibiting EAE development. Further work is necessary to confirm these findings and determine the mechanisms behind them.

## 5. Protection against Allergy and Asthma

The best evidence for *H. pylori-*mediated protection against extra-gastric disease comes from research on allergy and asthma. Although genetic predisposition is important, developing atopy is frequently associated with exposures in early life [114]. Protection against atopic disease may be mediated via a number of infectious organisms in the context of a diverse microbial microflora, rather than one simple component [115]. However, as the rate of *H. pylori* infection in childhood has declined in many developed countries, the prevalence of atopic disease in developed countries has increased markedly [116,117]. In one Finnish study for example, a 3-fold increase in the incidence of allergy was accompanied by a 30% decrease in *H. pylori* prevalence between 1973 and 1994 [118].

Multiple groups have shown that *H. pylori* infection is less common amongst those with atopy, and that *H. pylori-*infected adults and children are less likely to suffer from allergic asthma, rhinitis, food allergy, or have skin-prick allergen test sensitivity (reviewed in Table 1). Not all the studies agree, however the overwhelming evidence (supported by very large studies and meta-analyses) is supportive of a protective association. One of the key papers reported findings from a large population-based study of 7663 adults in the US National Health and Nutrition Examination Survey [32]. This confirmed a link between *H. pylori* negative infection status and having an early-life asthma diagnosis. People infected with CagA+ strains were even less likely to have had childhood asthma. Since not all strains express this virulence factor, this provided the first evidence that *H. pylori* could be driving these associations.

The strongest protective associations appear to be against childhood asthma [119,120], and age of the population being studied may be one of the reasons for contradictory findings. *H. pylori* infections are usually acquired in early childhood, a common age for onset of asthma [121]. Unfortunately current studies are limited by a paucity of data concerning the age of *H. pylori* acquisition and its relationship with age of asthma development. Other issues include the fact that asthma is not a uniform disease, being a manifest airway hyperresponsiveness to a variety of triggers including inhaled allergens, but also air pollutants, respiratory viruses, bacterial infections, and medications [25,122]. Asthma may also be worsened by aspiration of acidic gastric juice into the lungs [123,124]. The presence of GORD could therefore have an impact in some *H. pylori* studies.

*H. pylori* infections are usually established in early childhood [125], when the immune system is developing. The infection is known to stimulate a Th1 response in the gastric mucosa and also in peripheral blood [126,127]. Cytokines secreted by Th1 cells can counterbalance and suppress a Th2 response, which is the predominant T-helper subset associated with allergy [25]. In addition, infection with CagA+ *H. pylori* strains is reported to result in even greater Th1 responses [128] and reduced Th2 responses [129]. The *H. pylori* neutrophil-activating protein (HP-NAP) is an important Th1-promoting virulence factor, which has been shown to modulate Th2 responses in humans and mice [130,131,132,133]. When HP-NAP was administered via systemic or mucosal delivery to mice undergoing ovalbumin (OVA) allergen sensitization, this inhibited the development of lung eosinophilia, markedly reduced serum IgE levels, and there were lower bronchoalveolar Th2 cytokine concentrations [130]. *H. pylori* infection also influences the Th1/Th2 balance via effects on gastric hormones. When levels of somatostatin are reduced and gastrin production is increased, this also inhibits Th2 cytokine release and promotes Th1 responses [134].

The main immunological mechanism being investigated, however, is the *H. pylori-*mediated stimulation of Tregs. As previously mentioned (Section 3.2), these can act in a bystander manner to dampen immune responses such as those in asthma and allergy [135]. Th2 type inflammation in asthma is usually suppressed by Treg cells, and many current asthma therapies act by enhancing Treg responses. IL-10 is thought to play a major role in this, as it suppresses Th2 cell activity. It suppresses mast cell activation and cytokine production by mast cells and eosinophils. IL-10 also inhibits IgE production and promotes IgG4 production, an immunoglobulin balance thought to be protective against allergic responses. The role of the suppressive cytokine transforming growth factor β (TGF-β) is more complex, as apart from immunomodulation it is also involved in fibrosis and tissue remodeling in the airways [136,137,138].

Increased numbers of Tregs are present in the gastric mucosa and peripheral blood of *H. pylori-*infected patients [70,71,72,73,127]. *H. pylori* infection is also known to induce Treg responses in the gastric mucosa, peripheral blood and spleens of mice [72,139]. Stronger IL-10 and Treg responses are present in people with *cagA*+ strains [71,128], perhaps explaining the stronger protective associations between asthma and CagA+ infections [32]. CagA-dependent T cell priming in infected mice is also important for inducing Treg differentiation [140]. The fact that *H. pylori* stimulates a systemic Treg response supports the idea that such cells could have a more general immunoregulatory role.

In 2011, a paper from the group of Anne Müller provided the first mechanistic evidence that *H. pylori* infection could protect against allergic asthma in a mouse model [141]. Infected animals had significantly reduced airway hyperresponsiveness, measured by methacholine resistance, compared to uninfected controls. This was accompanied by a marked reduction in markers of asthma, including allergen-specific serum IgE, pulmonary infiltration of Th2 cells, Th17 cells and eosinophils, IL-5 and IL-13 in the lung lavage fluids, as well as a reduction in goblet cell metaplasia. These protective effects were strongest in mice that had been infected with a *cag*PAI+ strain of *H. pylori* as neonates, thus mimicking the human data where there was greater protection against childhood asthma, and in those infected with CagA+ strains [119]. The protection against asthma in mice was conferred by Tregs, since it could be induced by adoptive transfer of mesenteric lymph node cells from neonatally-infected donors, but not when the cells were depleted of Tregs prior to transfer. It was subsequently shown that *H. pylori* reprogrammes the differentiation of dendritic cells (DCs) to a tolerogenic phenotype, and these cells promote the differentiation of naïve T cells into Tregs. Such immature DCs (DC-SIGN^+^ HLA-DR^hi^ CD80^lo^ CD86^lo^) were also found in the infected human gastric mucosa [142]. More recently the group has shown that the regulatory cytokine IL-10 and CD103^+^CD11b^−^ dendritic cells are necessary to successfully protect against asthma with *H. pylori* in mice.

**Table 1:** Epidemiological evidence for and against *H. pylori-*mediated protection against allergy and asthma.

Studies that support protective associations		Studies that do not support protective associations	
Lower prevalence of *H. pylori* amongst atopic patients	[26,143]	No association between *H. pylori* seropositivity and atopy in children	[144]
Lower proportion of allergen-specific IgE positive amongst *H. pylori-*positive adults	[118]	No association between *H. pylori* exposure and measures of allergic disease or decline in lung function amongst randomly selected adults	[145]
Seropositivity to hepatitis A, *H. pylori* and *T. gondii* associated with a lower prevalence of atopy	[146]	Meta-analysis of 5 case-control studies for *H. pylori* and asthma risk found no protective association	[147]
Significant negative correlation between anti-*H. pylori* IgG and skin-prick allergen sensitivity	[148]	Non-significantly reduced *H. pylori* seropositivity amongst children with wheezing, but no associations with allergic rhinitis, atopic dermatitis, or asthma	[149]
*H. pylori* infection negatively associated with incidence of food allergy	[150]	No inverse relationship between *H. pylori* infection and adult asthma cases with peptic ulcer disease.	[151]
Lower prevalence of allergic rhinitis amongst *H. pylori-*positive adults	[152,153]		
Those colonised with CagA+ strains less likely to have ever had asthma compared to those without *H. pylori*, and less likely to have had asthma in childhood. Strongest protective association with asthma onset was in those younger than 5 years. Having a CagA+ infection significantly delayed asthma onset	[32,119,120]		
Reduced incidence of skin-prick allergen sensitivity in children positive for *H. pylori* by stool antigen test, in a longitudinal study	[154]		
Higher prevalence of allergic disease and a lower *H. pylori* infection rate among young adults	[155]		
Significantly reduced risk of atopy and asthma in those with *H. pylori* infection	[153,156,157,158]		

The *H. pylori* factors gamma-glutamyl transpeptidase (GGT) and vacuolating cytotoxin A (VacA) play an important role in the development of the Treg response. Intraperitoneal or intragastric delivery of purified VacA or recombinant GGT in mice could induce similar levels of protection against asthma as observed with the infection [159,160]. The effects of these components in humans must now be characterized to ensure that the mechanisms are clinically relevant, prior to investigating them as possible therapeutic agents.

## 6. Future Prospects and Conclusions

Investigating the impact of *H. pylori* on extra-gastric diseases is extremely challenging. The majority of the current evidence is based on cross-sectional epidemiological data, some of which may be flawed, and there is no way to assess cause and effect. Discrepancies between studies frequently arise from the use of different diagnostic methods, which may or may not be able to distinguish current from previous *H. pylori* infections. There is also a lack of adjustment for important confounding factors such as socioeconomic status, smoking, obesity and the effectiveness of eradication therapy. The genetic characteristics of both the bacterium and its host play an important role in determining the consequences of host-pathogen interactions, however this is extremely difficult to control for.

There are many remaining unknowns (see Table 2), and it is possible that some protective associations may ultimately not turn out to be driven by *H. pylori*. Perhaps *H. pylori* is merely a marker for other protective exposures. Alternatively, its observed effects could be mediated by modifying the complex microbiota of the gastro-intestinal tract rather than acting directly. To advance this field further, better-designed human studies must be carried out and ideally these should incorporate some way to assess cause and effect. This might be achieved by monitoring the long-term impact of *H. pylori* eradication, or with longitudinal studies, comparing disease severity over time in *H. pylori-*infected and uninfected patients. Such an approach cannot exclude the role of potential confounding exposures, however. The most direct way forward would be to perform clinical trials, monitoring effects arising from administering a *H. pylori* infection to volunteers. There have already been several human infection trials, however these have involved short-term periods of colonization and were not designed to investigate extra-gastric effects [33,161,162]. Data showing that particular strain types confer different levels of protection will be very informative, and we should establish whether immune-mediated protective effects in childhood can persist to later life, even when the infection has been eradicated. More animal model studies are needed to determine if the infection has a protective effect, and also to provide an understanding of the mechanisms. The results of these experiments should also be confirmed using human cells or tissue, to ensure that they are relevant to the clinical condition rather than mouse-specific mechanisms.

This field is difficult, but the benefits are potentially very far-reaching and rewarding. *H. pylori* is becoming less prevalent, and therefore the impact of this trend on the development of chronic immune and inflammatory disease must be assessed. As developing countries become more industrialised, loss of exposure to *H. pylori* could trigger a substantial increase in the prevalence of these debilitating diseases. In the future it may become possible to develop therapies based on *H. pylori* components. In the meantime however, strategies for *H. pylori* eradication should consider the possible health benefits conferred by this infection.

**Table 2:** Evidence gathered for *H. pylori-*mediated protection against extra-gastric diseases.

	Reduced prevalence of *H. pylori* in those with disease?	Documented effect of *H. pylori* eradication on disease?	Animal model data show *H. pylori* is protective?	Stronger protective effects from *cagA*+ strains?	Mechanistic data concerning protection?
**GORD**	yes	controversial	not done	yes	not done
**Ulcerative colitis**	yes	no	yes	unknown	yes
**Crohn’s disease**	yes	yes	not done	unknown	not done
**Coeliac disease**	yes	no	not done	unknown	not done
**Multiple sclerosis**	controversial	no	yes	unknown	not done
**Asthma**	yes	no	yes	yes	yes
